# Cardiovascular Safety of Abaloparatide in Postmenopausal Women With Osteoporosis: Analysis From the ACTIVE Phase 3 Trial

**DOI:** 10.1210/clinem/dgaa450

**Published:** 2020-07-13

**Authors:** Felicia Cosman, Linda R Peterson, Dwight A Towler, Bruce Mitlak, Yamei Wang, Steven R Cummings

**Affiliations:** 1 Department of Medicine, Columbia University, New York, New York; 2 Diabetic Cardiovascular Disease Center and Department of Medicine, Washington University, St Louis, Missouri; 3 Department of Internal Medicine, UT Southwestern Medical Center, Dallas, Texas; 4 Clinical Development, Radius Health, Inc., Waltham, Massachusetts; 5 Biostatistics, Radius Health, Inc., Waltham, Massachusetts; 6 San Francisco Coordinating Center, Sutter Health, California; University of California, San Francisco, California

**Keywords:** abaloparatide, blood pressure, MACE, cardiovascular, heart rate, osteoporosis

## Abstract

**Context:**

Abaloparatide is a US Food and Drug Administration-approved parathyroid hormone-related peptide analog for treatment of osteoporosis in postmenopausal women at high risk of fracture.

**Objectives:**

We assessed the cardiovascular safety profile of abaloparatide.

**Design:**

Review of heart rate (HR), blood pressure (BP), and cardiovascular-related adverse events (AEs), including major adverse cardiovascular events (MACEs) and heart failure (HF) from: (a) ACTIVE (NCT01343004), a phase 3 trial that randomized 2463 postmenopausal women with osteoporosis to abaloparatide, teriparatide, or placebo for 18 months; (b) ACTIVExtend (NCT01657162), where participants from the abaloparatide and placebo arms received alendronate for 2 years; and (c) a pharmacology study in 55 healthy adults.

**Results:**

Abaloparatide and teriparatide transiently increased HR relative to placebo. Following first dose, mean (standard deviation [SD]) HR change from pretreatment to 1 hour posttreatment was 7.9 (8.5) beats per minute (bpm) for abaloparatide, 5.3 (7.5) for teriparatide, and 1.2 (7.1) for placebo. A similar pattern was observed over subsequent visits. In healthy volunteers, HR increase resolved within 4 hours. The corresponding change in mean supine systolic and diastolic BP 1 hour posttreatment was –2.7/–3.6 mmHg (abaloparatide), –2.0/–3.6 (teriparatide), and –1.5/–2.3 (placebo). The percentage of participants with serious cardiac AEs was similar among groups (0.9%-1.0%). In a post hoc analysis, time to first incidence of MACE + HF was longer with abaloparatide (*P = *0.02 vs placebo) and teriparatide (*P = *0.04 vs placebo).

**Conclusions:**

Abaloparatide was associated with transient increases in HR and small decreases in BP in postmenopausal women with osteoporosis, with no increase in risk of serious cardiac AEs, MACE, or HF.

In addition to the central role of parathyroid hormone-related peptide (PTHrP) in bone and cartilage development, PTH/PTHrP signaling is important in cardiovascular development and functioning (1-3). PTHrP acts in a paracrine fashion to regulate local vascular tone, and it has been known for decades that acute administration of PTH and PTHrP causes vasodilatation of the coronary, renal, and other vascular beds (3-5). In healthy volunteers, infusion of PTHrP increased heart rate (HR) and renal plasma flow without a significant effect on other hemodynamic parameters (arterial pressure, electromechanical systole corrected for HR [QS_2c_]) (2). In preclinical models, PTHrP improved the contractile function of myocardium subjected to ischemia-reperfusion (6), and PTHrP protected cultured cardiomyocytes from oxidative stress (7).

Abaloparatide is a synthetic analog of PTHrP and selective agonist of the PTH type 1 receptor (PTHR1) that is US Food and Drug Administration (FDA) approved for the treatment of osteoporosis in postmenopausal women at high risk for fracture (8). The efficacy and safety of abaloparatide was established in the Abaloparatide Comparator Trial in Vertebral Endpoints (ACTIVE) trial, in which 18 months of treatment with abaloparatide produced significantly greater reductions in risk of new vertebral and nonvertebral fractures versus placebo and significantly greater reductions in risk of major osteoporotic fracture versus placebo and teriparatide (9). In the extension study (ACTIVExtend), where participants from the abaloparatide and placebo groups were treated with oral alendronate (10, 11), reductions in fracture risk and increased bone mineral density increments were sustained.

There were no differences in adverse events (AEs) between the treatment groups in ACTIVE. Nonserious AEs leading to study-drug discontinuation occurred more frequently with abaloparatide (nausea [1.6%], dizziness [1.2%], headache [1.0%], and palpitations [0.9%]) compared with placebo or open-label teriparatide (9). During the first 6 months of treatment, a slightly greater number of participants withdrew from the abaloparatide group compared with the teriparatide or placebo groups, but from 6 months on, discontinuation was similar among groups.

At the request of a regulatory agency, a post hoc analysis of major adverse cardiovascular events (MACE) and MACE plus heart failure (HF) was conducted in ACTIVE and was further assessed during ACTIVExtend. The objective of the current analysis was to assess the cardiovascular safety profile of abaloparatide using HR, blood pressure (BP) measurements, and AEs potentially associated with changes in HR and BP from ACTIVE and to determine the frequency of MACE after abaloparatide treatment concluded. Further information on HR changes was examined in a clinical pharmacology study conducted in 55 healthy women and men.

## Materials and Methods

### ACTIVE and ACTIVExtend study design and participants

ACTIVE was a randomized, double-blind, placebo- and active-controlled, multicenter, phase 3 study (clinicaltrials.gov identifier NCT01343004). ACTIVExtend was a 24-month, open-label extension of ACTIVE (clinicaltrials.gov identifier NCT01657162). Details of ACTIVE and ACTIVExtend have been previously reported (9-11). Participants in ACTIVE were postmenopausal women between the ages of 49 and 86 years with osteoporosis defined by bone mineral density and prior fracture history, as previously described. Women were randomized to daily subcutaneous abaloparatide 80 µg, matching placebo, or open-label daily subcutaneous teriparatide 20 µg for 18 months. Concomitant medications such as statins, aspirin, or antihypertensives were allowed if the dose was stable at entry. Every effort was made to maintain a stable dose during study participation. After an approximately 1-month treatment-free period for reconsent, eligible participants who had been randomized to either abaloparatide or placebo in ACTIVE were enrolled in ACTIVExtend and transitioned to open-label alendronate 70 mg once-weekly for 24 months.

The studies were conducted according to the recommendations of Good Clinical Practice and the Declaration of Helsinki revised edition (Seoul 2008 and Tokyo 2004) and were approved by the ethics committee at every participating study site. All participants provided written informed consent.

### Cardiovascular-related eligibility criteria

On visit 1 during the screening period, BP measurements and electrocardiograms (ECGs) were obtained. To be enrolled, participants were required to have sitting or supine systolic BP ≥ 100 and ≤ 155 mmHg, diastolic BP ≥ 40 and ≤ 95 mmHg, and HR ≥ 45 and ≤ 100 beats per minute (bpm). Based on single measurements at screening, women with a decrease of ≥ 20 mmHg in systolic BP or ≥ 10 mmHg in diastolic BP based on supine to standing maneuver, or any symptomatic hypotension were excluded. Additionally, women with clinically significant abnormalities or QTc > 470 msec (Bazett correction) by resting 12-lead ECG were excluded, as were women with cardiovascular disease to the extent that it would affect patient safety or interfere with study interpretation as determined by individual investigators.

### Outcomes

The primary endpoints from ACTIVE and ACTIVExtend have been previously reported (9-11). Cardiovascular outcomes evaluated a priori were changes in HR, supine and standing BP, and the percentages of participants with serious cardiac treatment-emergent AEs. In ACTIVE, HR was assessed using ECGs at baseline, before, and 1 hour after study medication administration; at 6 study visits during the first year of the study; and at the end of treatment (18 months). BP was measured at each visit prior to dosing and again 1 hour after administration of study medication after participants were supine for 5 minutes and then again after standing for 3 minutes. Medical dictionary for regulatory activities (MedDRA [v17.1]) preferred terms for AEs potentially associated with an HR increase were palpitations, tachycardia, dizziness, and nausea.

Cardiovascular outcomes evaluated post hoc were the percentages of participants with AEs potentially associated with changes in HR and BP, MACE, MACE + HF, and time to first incidence of MACE and MACE + HF. Clinical judgement was used to group AEs that were increased with abaloparatide and teriparatide and were potentially associated with changes in HR or BP. Serious cardiac AEs were presented separately and not double counted. MACEs included nonfatal myocardial infarction, nonfatal stroke, and cardiovascular death. For the MACE analyses, study sites were recontacted to request information regarding cardiovascular events; however, insufficient information was available to conduct an adjudication for many cases. Therefore, standardized MedDRA queries (SMQs) for medical history and treatment-emergent AEs of interest were performed. As this approach also led to events that could not be adjudicated, we used MedDRA preferred terms for MACE within the System Organ Class groupings of Cardiac Disorders, Nervous System Disorders, and Vascular Disorders to identify MACE. These data were then reviewed by a consulting cardiologist as well as by other experienced researchers without knowledge of the treatment group assignments. Clinical AEs that most clearly represented MACE + HF were identified and included in the analyses. For serious cardiac AEs, AEs associated with HR increase, MACE, and MACE + HF, each participant was counted once for the same class grouping and the same preferred term. A sensitivity analysis using SMQ categorizations was used to assess the robustness of our approach.

### Study in healthy volunteers

Because HR was assessed only predose and 1 hour postdose in ACTIVE, information from a clinical pharmacology study of abaloparatide conducted in 55 healthy nonsmoking volunteers (32 men, 23 women; aged 18-54 years) was included in this report of cardiovascular safety with abaloparatide. In this study, HR was assessed by ECG following a single dose of abaloparatide or placebo at 15 minutes, 30 minutes, 45 minutes, 1 hour, and 1.5, 2, 2.5, 4, 6, 8, and 12 hours.

### Data analysis

All outcomes from ACTIVE were analyzed using the safety population, defined as all participants who received at least 1 dose of study medication (all participants as treated). HR and BP were assessed by descriptive statistics (mean, standard deviation [SD], median, maximum, minimum), and *P* values between pairs of treatment groups were calculated using analysis of variance or a mixed-effect repeated-measure model for longitudinal data. In addition, maximal predose to postdose changes over 6 visits during the first 12 months in HR and BP (considering supine systolic and diastolic BP, standing systolic and diastolic BP measurements separately) were assessed for each individual and a “mean maximal HR and BP change” was calculated. Pearson correlation coefficients were calculated to determine if there were relationships between BP and HR changes.

Categorical data from ACTIVE and ACTIVExtend are reported as number and percentage of participants. Time to first incidence and absolute risk reduction (%) of MACE or MACE + HF were determined by Kaplan-Meier estimates, and 95% confidence intervals (CIs) for Kaplan-Meier estimates used a log-log transformation and for absolute risk reduction used standard error obtained by the Greenwood formula with the normal approximation. After confirming the proportionality assumption, a Cox proportional hazards model was used to calculate the hazard ratio with placebo or teriparatide as the reference. *P* values were calculated from the log-rank test. Age-adjusted MACE incidence rate per 100 person-years was calculated for abaloparatide versus abaloparatide/alendronate and for placebo versus placebo/alendronate.

In the healthy volunteers study, placebo-adjusted HR change from baseline for abaloparatide and 90% CI was calculated from the difference of least squares means between abaloparatide and placebo at each time point based on analysis of covariance model. Covariates included baseline HR, treatment, time, treatment and time interaction, and crossover design factors of period and sequence. The model allowed the intercept parameter to be random.

Statistical analyses were conducted using SAS version 9.4 (Cary, NC).

## Results

### Participants

The ACTIVE safety population included 2460 participants, 1133 of whom were followed in ACTIVExtend. Approximately 5% of study participants had a history of cardiac disease and 4% a history of cerebrovascular disease. In addition, a large number of participants had cardiovascular risk factors at baseline (66% abaloparatide, 67% teriparatide, and 67% placebo). The proportions of participants with prior history of cardiac and cerebrovascular disease, use of cardioactive medications, and cardiovascular risk factors at baseline were balanced among groups (Table 1).

**Table 1.** History of cardiovascular risk factors at baseline in ACTIVE

	Abaloparatide N = 822	Teriparatide N = 818	Placebo N = 820	Total N = 2460
Age, mean (SD), y	68.9 (6.50)	68.8 (6.57)	68.7 (6.48)	68.8 (6.51)
Any cardiovascular risk factors at baseline, n (%)	544 (66.2)	544 (66.5)	551 (67.2)	1639 (66.6)
Hypertension	374 (45.5)	407 (49.8)	383 (46.7)	1164 (47.3)
Dyslipidemia	370 (45.0)	341 (41.7)	362 (44.1)	1073 (43.6)
Diabetes	94 (11.4)	90 (11.0)	88 (10.7)	272 (11.1)
Coronary disease^a^	50 (6.1)	35 (4.3)	34 (4.1)	119 (4.8)
Cerebrovascular disease^b^	39 (4.7)	38 (4.6)	25 (3.0)	102 (4.1)
Atrial fibrillation/flutter	5 (0.6)	6 (0.7)	6 (0.7)	17 (0.7)
Revascularization	4 (0.5)	3 (0.4)	5 (0.6)	12 (0.5)
Any cardiovascular medication at baseline, n (%)	468 (56.9)	461 (56.4)	449 (54.8)	1378 (56.0)
Antihypertensives	376 (45.7)	377 (46.1)	373 (45.5)	1126 (45.8)
Hypolipidemics	216 (26.3)	201 (24.6)	201 (24.5)	618 (25.1)
Platelet aggregation inhibitors	117 (14.2)	85 (10.4)	94 (11.5)	296 (12.0)

^a^Coronary disease includes coronary artery disease, coronary artery insufficiency, myocardial infarction, and acute myocardial infarction.

^b^Cerebrovascular disease includes cerebral infarction, cerebral ischemia, cerebral thrombosis, cerebrovascular accident, cerebrovascular disorder, embolic stroke, intracranial hemorrhage, hemorrhagic stroke, ischemic stroke, and transient ischemic attack.

Abbreviations: SD, standard deviation; y, years.

### Heart rate

**Figure 1.**
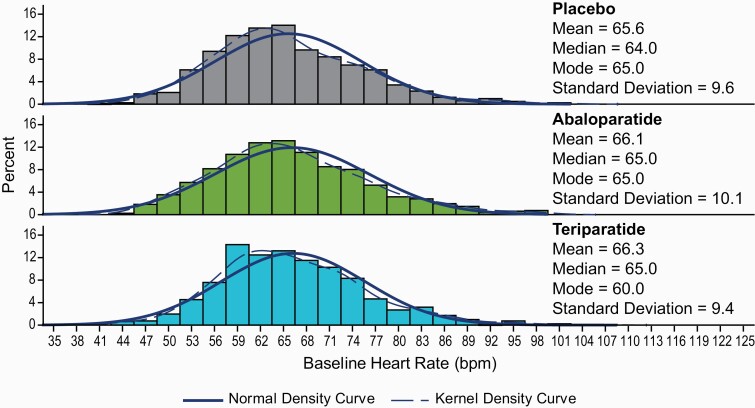
Baseline heart rate by treatment group in ACTIVE. bpm, beats per minute.

Across study visits, mean (SD) baseline HR, based on 12-lead ECG, was 66.1 (10.1) bpm, 66.3 (9.4) bpm, and 65.6 (9.6) bpm, in the abaloparatide, teriparatide, and placebo groups, respectively (Fig. 1). Baseline mean predose HR did not differ over time during the trial in any of the 3 groups. The mean (SD) HR change from pretreatment to 1 hour posttreatment on day 1 was 7.9 (8.5) bpm, 5.3 (7.5) bpm, and 1.2 (7.1) bpm for abaloparatide, teriparatide, and placebo, respectively (*P < *0.0001 for abaloparatide and teriparatide vs placebo; *P < *0.05 for abaloparatide vs teriparatide) (Table 2). Similar changes were observed at subsequent visits during the first year of treatment.

**Table 2.** Mean change in heart rate from predose to 1 hour postdose by visit in ACTIVE

	Visit	Abaloparatide^a^	Teriparatide^a^	Placebo
Heart rate change (bpm) mean (SD)	Day 1	7.9 (8.5)	5.3 (7.5)	1.2 (7.1)
	Month 1	7.3 (8.6)	5.1 (7.4)	1.6 (6.7)
	Month 3	7.4 (8.2)	5.3 (7.3)	1.5 (6.7)
	Month 6	6.8 (8.1)	6.0 (7.4)	1.2 (6.6)
	Month 9	7.2 (8.2)	5.5 (7.3)	1.9 (6.6)
	Month 12	7.2 (8.4)	5.9 (7.4)	1.8 (6.7)

^a^ *P < *0.05 vs placebo.

Abbreviations: bpm, beats per minute; SD, standard deviation.

**Figure 2.**
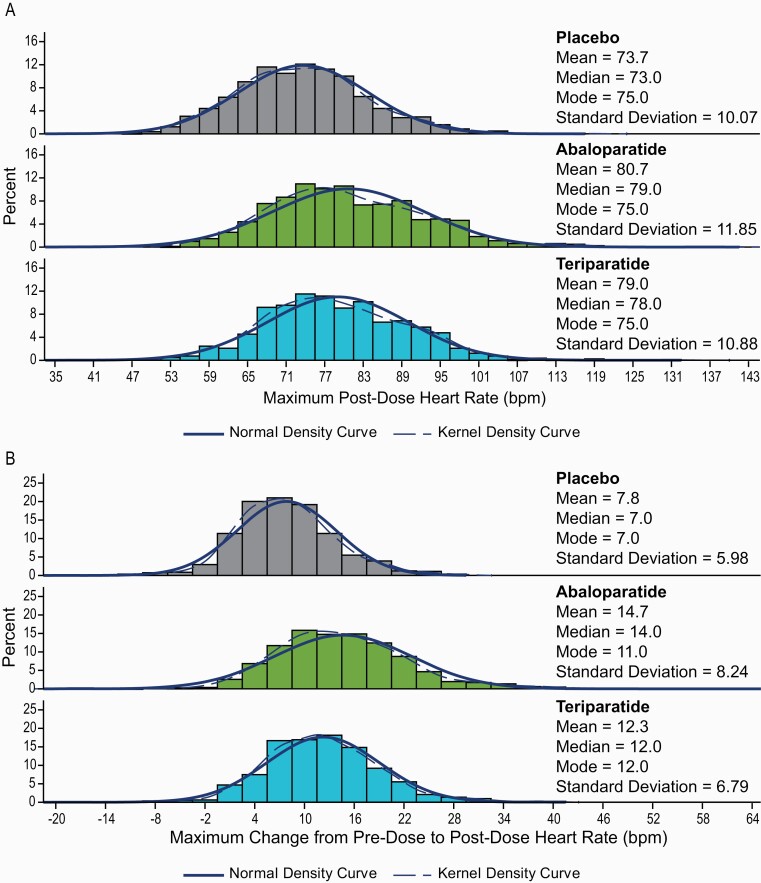
Distribution of maximum heart rate in ACTIVE. **A)** 1 hour postdose by treatment group. **B**) Change from pre- to postdose. bpm, beats per minute.

**Figure 3.**
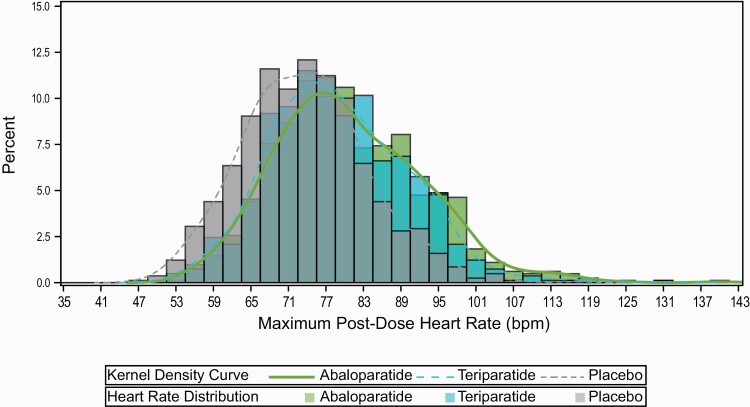
Overlap of distribution of maximum 1-hour postdose heart rate for abaloparatide and teriparatide in ACTIVE. bpm, beats per minute.

The maximal 1 hour postdose HR across all study visits was also assessed for each participant during the first year of the study; the mean (SD) maximum 1-hour postdose HR was 80.7 (11.9) bpm for abaloparatide and 79.0 (10.9) bpm for teriparatide, compared with 73.7 (10.1) bpm in the placebo group (*P < *0.0001 for abaloparatide and teriparatide vs placebo and *P < *0.01 for abaloparatide vs teriparatide) (Fig. 2A and Fig. 3). There was approximately a 95% overlap in maximum 1-hour postdose HR between participants in the abaloparatide and teriparatide groups (Fig. 3).

The mean maximal change from predose to 1-hour postdose HR was 14.7 (8.2) bpm in the abaloparatide group, 12.3 (6.8) bpm with teriparatide and 7.8 (6.0) bpm with placebo; the changes were greater for both treatment groups compared with placebo and for abaloparatide compared with teriparatide, *P < *0.001 (Fig. 2B).

The percentage of participants with a transient HR ≥ 105 bpm was 3.0% (25/821) with abaloparatide, 1.6% (13/817) with teriparatide, and 0.2% (2/819) with placebo; and with a transient HR between 117 and 120 bpm was 0.5% (4/821) with abaloparatide, 0.4% (3/817) with teriparatide, and 0.1% (1/819) with placebo. One participant in the abaloparatide group had a single visit where 90% of the target age-adjusted maximum HR (maximum = 220 bpm minus age) was exceeded at month 3. The participant had a prior history of hypertension and did not experience any cardiac AEs during ACTIVE.

**Figure 4.**
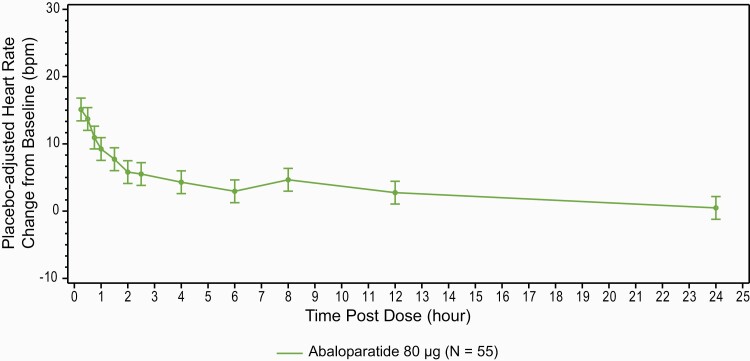
Heart rate change over 24 hours following a single administration of abaloparatide versus placebo in healthy participants (90% CI). 5 bpm equals expected variability of resting heart rate in healthy participants. bpm, beats per minute; CI, confidence interval.

In the healthy volunteer study, HR was highest 15 minutes after dosing, the earliest time assessed, and then declined. Approximately 2.5 hours postdose, the mean placebo-adjusted HR difference from baseline was < 5 bpm. From 4 to 12 hours postdose, the mean placebo-adjusted HR ranged from 2.2 to 4.0 bpm higher than baseline (Fig. 4).

### Blood pressure

There were small (1-3 mm Hg), but statistically significant, decreases in mean supine and standing systolic and diastolic BP 1 hour after dosing between abaloparatide and teriparatide groups and placebo after the first dose (Table 3) and during all visits over the first year.

**Table 3.** Change in blood pressure from predose to 1 hour postdose

Visit		Abaloparatide N = 822	Teriparatide N = 818	Placebo N = 820
Day 1 Mean (SD) (mmHg)	Supine systolic	–2.7 (11.6)^a^	–2.0 (10.1)	–1.5 (10.8)
	Supine diastolic	–3.6 (7.2)^a^	–3.6 (6.9)^a^	–2.3 (6.9)
	Standing systolic	–3.4 (13.4)^a^	–2.0 (11.3)^a^	–0.7 (11.7)
	Standing diastolic	–4.3 (7.7)^a^	–3.5 (7.0)^a^	–2.1 (6.7)
Maximal drop in BP during the 1st year visits Mean (SD) (mmHg)	Supine systolic	–14.7 (12.1)^a^	–14.7 (9.8)^a^	–13.6 (9.8)
	Supine diastolic	–11.4 (7.3)^a^	–11.2 (6.2)	–10.7 (6.4)
	Standing systolic	–15.9 (13.3)^a^	–15.1 (11.5)^a^	–13.6 (10.7)
	Standing diastolic	–11.6 (7.9)^a^	–11.3 (6.5)^a^	–10.4 (6.5)

^a^ *P < *0.05 vs placebo.

Abbreviations: BP, blood pressure; SD, standard deviation.

The mean maximal decrease in predose to 1-hour postdose BP during the first year of the study was slightly higher (1-2 mmHg) in the abaloparatide and teriparatide groups compared with placebo (*P < *0.05).

There were weak correlations between change in HR and BP in all groups (r values between 0.003 and 0.26). The proportion of women with at least a 20 mmHg decline in systolic BP from supine to standing or at least a 10 mmHg decrease in diastolic BP from supine to standing 1 hour postdose was 17.1%, 15.5%, and 16.4% in the abaloparatide, teriparatide, and placebo groups, respectively (*P > *0.4 for all pairwise comparisons).

### Cardiac AEs reported in ACTIVE

The percentage of participants with treatment-emergent AEs potentially associated with changes in HR and BP (eg, palpitations, tachycardia, dizziness, and nausea) during ACTIVE was larger with abaloparatide and teriparatide and reported more frequently with both active treatments compared with placebo; few were serious AEs (Table 4).

**Table 4.** Treatment-emergent adverse events potentially associated with heart rate increases in ACTIVE

Preferred Term, n (%)	Abaloparatide N = 822	Teriparatide N = 818	Placebo N = 820
**Any TEAE** ^**a**^	165 (20.1)	106 (13.0)	74 (9.0)
Palpitations	42 (5.1)	13 (1.6)	3 (0.4)
Tachycardia	11 (1.3)	6 (0.7)	3 (0.4)
Dizziness	82 (10.0)	60 (7.3)	50 (6.1)
Nausea	68 (8.3)	42 (5.1)	25 (3.0)
**Any serious TEAE** ^**a**^	1 (0.1)	3 (0.4)	1 (0.1)
Serious palpitations	1 (0.1)	1 (0.1)	0
Serious tachycardia	0	0	0
Serious dizziness	0	2 (0.2)	1 (0.1)
Serious nausea	0	0	0
**Any TEAE** ^**a**^ **leading to discontinuation**	27 (3.3)	11 (1.3)	5 (0.6)
Palpitations	7 (0.9)	0	1 (0.1)
Tachycardia	2 (0.2)	0	0
Dizziness	10 (1.2)	8 (1.0)	3 (0.4)
Nausea	13 (1.6)	3 (0.4)	2 (0.2)
**Any serious TEAE** ^**a**^ **leading to discontinuation**	0	1 (0.1)	0
Serious palpitations	0	0	0
Serious tachycardia	0	0	0
Serious dizziness	0	1 (0.1)	0
Serious nausea	0	0	0

^a^Treatment-emergent was defined as any AE that occurred on or after the day of administration of the first dose of study drug, any event that was considered drug-related (the causality was either possible or probable) regardless of the start date of the event, or any event that was present at baseline but worsened in severity or was subsequently considered drug-related by the Investigator.

Abbreviations: AE, adverse event; TEAE, treatment-emergent adverse event.

The percentage of participants who discontinued study drug in ACTIVE because of treatment-emergent AEs potentially associated with changes in HR and BP was 3.3% (27/822) in the abaloparatide group, 1.3% (11/818) in teriparatide, and 0.6% (5/820) in placebo groups.

Overall, 9.9% (81/822), 6.8% (56/818), and 6.1% (50/820) in the abaloparatide, teriparatide, and placebo groups, respectively, had at least 1 AE leading to discontinuation of study drug.

The percentage of participants with serious cardiac AEs evaluated in ACTIVE was similar in the abaloparatide (1.0%; 8/822), teriparatide (1.0%; 8/818), and placebo (0.9%; 7/820) groups. In the abaloparatide group, 0.7% (6/822) reported syncope, compared with 1% (8/818) for teriparatide, and 1.1% (9/820) in placebo groups.

### MACE and MACE + heart failure

**Figure 5.**
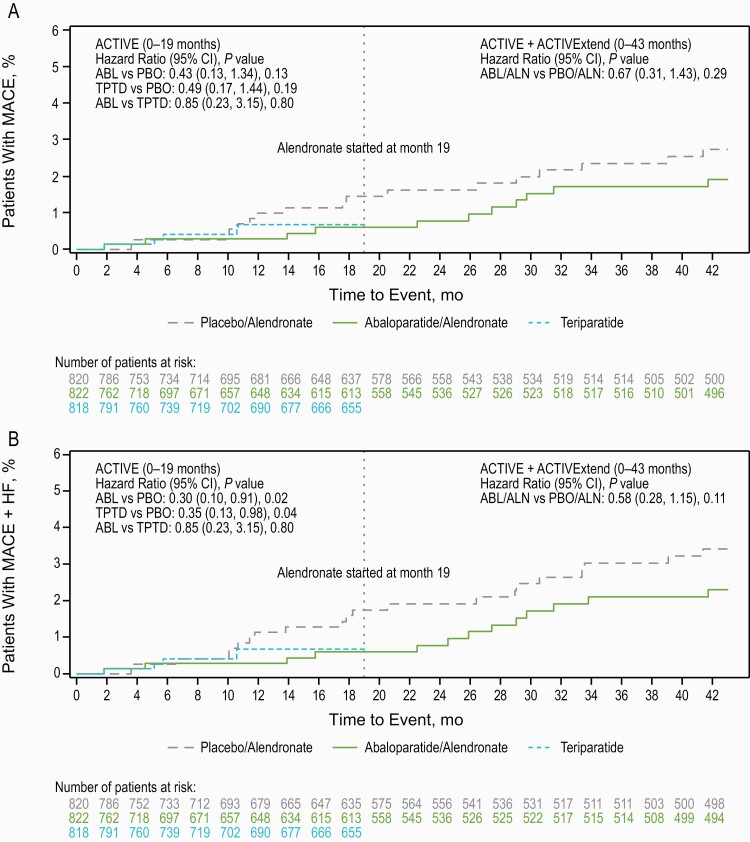
Time to first incidence of MACE and MACE + HF in ACTIVE and ACTIVExtend. **A**) ACTIVE and ACTIVExtend MACE; **B**) ACTIVE and ACTIVExtend MACE + HF. ABL, abaloparatide; ALN, alendronate; HF, heart failure; MACE, major adverse cardiovascular event; mo, months; PBO, placebo; TPTD, teriparatide.

During ACTIVE, few participants experienced MACE. The percentage of participants with MACE was 0.5% in the abaloparatide group, 0.6% in the teriparatide group, and 1.2% in the placebo group (Table 5). The percentage of participants with MACE + HF was 0.5% in the abaloparatide group, 0.6% in the teriparatide group, and 1.7% in the placebo group (Table 5). The sensitivity analysis using SMQ groupings (based on pooling the analysis) was generally consistent with the identification of MACE and MACE + HF using our approach. The time to first incidence of MACE was not significantly different with either abaloparatide or teriparatide versus placebo in ACTIVE (Fig. 5A). The absolute risk reduction (95% CI) and hazard ratio (95% CI) were –0.85% (–1.91 to 0.21) and 0.42 (0.13-1.3) (*P = *0.13) for abaloparatide versus placebo and –0.76% (–1.83 to 0.31) and 0.49 (0.17-1.44) (*P = *0.19) for teriparatide versus placebo.

**Table 5.** Major adverse cardiovascular events and major adverse cardiovascular events plus heart failure events in ACTIVE

Preferred Term, n (%)	Abaloparatide N = 822	Teriparatide N = 818	Placebo N = 820
ACTIVE (0-18 months) MACE	4 (0.5)	5 (0.6)	10 (1.2)
Nonfatal stroke or MI	3 (0.4)	4 (0.5)	8 (1.0)
Acute MI	0	1 (0.1)	0
Cerebrovascular accident	0	1 (0.1)	3 (0.4)
Hemorrhage intracranial	1 (0.1)	0	0
Ischemic stroke	0	0	3 (0.4)
Lacunar infarction	1 (0.1)	1 (0.1)	0
MI	1 (0.1)	1 (0.1)	2 (0.2)
Fatal CV event/sudden death	1 (0.1)	1 (0.1)	3 (0.4)
Aortic dissection	0	0	1 (0.1)
Cardiorespiratory arrest	0	1 (0.1)	0
MI	0	0	1 (0.1)
Myocardial ischemia	1 (0.1)	0	0
Sudden death	0	0	1 (0.1)
ACTIVE (0-18 months) MACE + HF	4 (0.5)	5 (0.6)	14 (1.7)
Nonfatal cardiac failure	0	1 (0.1)	4 (0.5)
Cardiac failure	0	1 (0.1)	3 (0.4)
Cardiac failure chronic	0	0	1 (0.1)
Cardiac failure congestive	0	0	1 (0.1)

CV, cardiovascular; HF, heart failure; MACE, major adverse cardiovascular event; MI, myocardial infarction.

The time to first incidence of MACE + HF was significantly longer with abaloparatide versus placebo (*P = *0.02). The absolute risk reduction (95% CI) and hazard ratio (95% CI) for MACE + HF was –1.43 (–2.63 to –0.23) and 0.30 (0.10-0.91), respectively, with abaloparatide versus placebo. The time to first incidence for MACE + HF was also longer for teriparatide versus placebo (*P = *0.04). The absolute risk reduction (95% CI) and hazard ratio (95% CI) was –1.34% (–2.54 to –0.13) and 0.35 (0.13-0.98), respectively for teriparatide versus placebo.

### ACTIVExtend

After abaloparatide treatment was discontinued during the 24-month ACTIVExtend trial, the percentage of participants with MACE (1.3% abaloparatide/alendronate; 1.2% placebo/alendronate) and MACE + HF (1.6% abaloparatide/alendronate; 1.6% placebo/alendronate) was similar between groups (Table 6). The time to first incidence of MACE and MACE + HF was also similar between the abaloparatide/alendronate and placebo/alendronate groups in ACTIVExtend (Fig. 5B).

**Table 6.** Major adverse cardiovascular events and major adverse cardiovascular events plus heart failure events in ACTIVExtend

Preferred Term, n (%)	Placebo/ Alendronate N = 580	Abaloparatide/ Alendronate N = 553
ACTIVExtend (0-24 months) MACE	7 (1.2)	7 (1.3)
Nonfatal stroke or MI	7 (1.2)	7 (1.3)
Acute MI	3 (0.5)	1 (0.2)
Basal ganglia stroke	0	1 (0.2)
Cerebral thrombosis	0	1 (0.2)
Cerebrovascular accident	2 (0.3)	0
Ischemic stroke	1 (0.2)	2 (0.4)
Left ventricular failure	0	1 (0.2)
MI	2 (0.3)	1 (0.2)
Fatal CV event/sudden death	2 (0.3)	0
Acute MI	1 (0.2)	0
MI	1 (0.2)	0
ACTIVExtend (0-24 months) MACE + HF	9 (1.6)	9 (1.6)
Nonfatal cardiac failure	2 (0.3)	3 (0.5)
Cardiac failure	0	2 (0.4)
Cardiac failure chronic	0	1 (0.2)
Cardiac failure congestive	2 (0.3)	0

Abbreviations: CV, cardiovascular; HF, heart failure; MACE, major adverse cardiovascular event; MI, myocardial infarction.

Additionally, the age-adjusted MACE incidence rate per 100 patient-years was not significantly different after transitioning from abaloparatide to alendronate (rate difference abaloparatide minus abaloparatide/alendronate, –0.36; *P = *0.29) or from placebo to alendronate (rate difference placebo/alendronate minus placebo, –0.14; *P = *0.72).

## Discussion

We examined the cardiovascular profile of abaloparatide, compared with treatment with placebo and teriparatide in ACTIVE. Treatment with abaloparatide and teriparatide was associated with a transient increase in HR, compared with placebo. The mean maximal HR observed 1 hour after dosing was slightly greater for abaloparatide than for teriparatide. In the healthy volunteer study, the transient increase in HR resolved within 2.5 to 4 hours. BP was modestly lower for abaloparatide and teriparatide groups 1 hour after the first dose and during the first year of the study with a mean maximal difference of 1 to 3 mmHg versus placebo.

Neither treatment with abaloparatide or teriparatide was associated with an increase in serious cardiac AEs. In fact, the frequency of MACE and MACE + HF was numerically lower and the time to first incidence of MACE + HF events was significantly longer with abaloparatide and teriparatide compared with placebo. Results from the ACTIVExtend trial, in which participants from the abaloparatide and placebo groups from ACTIVE were switched to alendronate for 24 months, indicated that the frequency of MACE and time to first incidence of MACE became similar between groups once abaloparatide was discontinued.

Abaloparatide and teriparatide appear to increase HR by a similar mechanism. The PTHR1 is expressed and active within arterial smooth muscle cells, and at lower levels within the heart, and PTHrP acts in a paracrine fashion in the regulation of local vascular contractile tone (3, 5). In a study of healthy volunteers, PTHrP infusion resulted in an increase in HR with no significant effect on BP as assessed by brachial cuff (2). Preclinical data from dogs have demonstrated that teriparatide induces a dose-dependent increase in HR (12). In addition, circulating PTHrP levels have been identified as being elevated in patients with atrial fibrillation, suggestive of an adaptive physiologic/compensatory role to reduce afterload with the reduced “atrial kick” and cardiac output that arises in this setting (13). As such, there has been insufficient data published on the impact of PTH1r agonists on HR and cardiovascular physiology in humans. In the pivotal Fracture Prevention Trial of teriparatide, no changes in pre-injection HR were observed, but postinjection HR was not reported (14). Additionally, electrophysiologic studies of sinus node and Purkinje fibers showed that PTH increased automaticity associated with changes in the hyperpolarization-activated current or funny current (If) (15). These observations suggest that the transient increase of HR following abaloparatide and teriparatide administration may be the result of an effect on the sinus node. While an HR response to vasodilation cannot be excluded, there was no meaningful relationship observed between decrease in BP and increase in HR.

Although there were exclusions for significant cardiovascular disease in ACTIVE, a large and similar proportion in both the active treatment and placebo groups had a history of cardiac disorders and/or were taking concomitant cardioactive medications. While predose HR was similar across groups, it is of interest that the average HRs were somewhat lower than typical for the average US population, in part because a high proportion of participants were outside the United States where average HRs are lower. The lower rate of MACE and significantly longer time to first incidence of MACE + HF with abaloparatide and teriparatide compared with placebo warrants further investigation. Indeed, there is a limited body of evidence suggesting a potential cardioprotective effect of PTHrP in ischemia reperfusion injury (7). In addition, studies in preclinical models have demonstrated potential vasoprotective actions of paracrine PTHrP/PTHR1 in control of vascular tone, limiting vascular fibrosis, and homeostatic regulation of renal end-organ perfusion (3, 16).

A recent meta-analysis suggested that PTH analogs have no impact on cardiovascular risk and overall mortality (17). Prior studies with other osteoporosis agents have highlighted the potential interaction of the bone and cardiovascular system. The development of odanacatib, a cathepsin K inhibitor, was discontinued due to an apparent increase in the risk of cerebrovascular disease observed in the pivotal phase 3 study and an increase in the composite MACE endpoint in the phase 3 extension trial of odanacatib (18). Romosozumab, a monoclonal antibody that binds and inhibits sclerostin (19), was approved by the FDA for the treatment of postmenopausal women at high risk for fracture in April 2019. The Fracture Study in Postmenopausal Women with Osteoporosis (FRAME) and Active-Controlled Fracture Study in Postmenopausal Women with Osteoporosis at High Risk (ARCH) trials were phase 3, randomized, double-blind, controlled trials of romosozumab (20, 21). There was no effect of romosozumab versus placebo on the frequency of adjudicated MACE after 12 months of treatment in the FRAME trial (0.8% and 0.8%, respectively), but there was a difference between the romosozumab and alendronate groups for adjudicated MACE in the ARCH trial (2.0% and 1.1%, respectively) (19). The reason for the differences between groups for cardiovascular events in ARCH and FRAME is unknown, but the authors of the ARCH trial suggested the possibility that bisphosphonates might reduce the risk of cardiovascular events (22). We did not observe any decline in MACE upon transition from abaloparatide or placebo to alendronate during ACTIVExtend. While it remains to be confirmed, the difference in MACE between romosozumab and alendronate over 1 year in the ARCH study might be due to chance (23).

There are limitations to our study. First, the post hoc analysis of MACE and HF is limited since ACTIVE was not designed and did not have sufficient power to evaluate MACE and the number of these events was low. BP measurements were only made after medication administration at the 1-hour time point, so we do not have data on any possible earlier or later effects. Due to lack of access to detailed participant-level information, formal adjudication of potential cardiovascular events was not possible; however, qualifying events were determined by a cardiologist and experienced investigators in a blinded fashion. This, in addition to the sensitivity analysis using SMQ groupings, which was consistent with our results, provides credibility to the analysis. Although prospective formal adjudication of MACE is optimal, recently, results from the STABILITY trial indicated that primary cardiovascular outcomes were consistent between investigator-reported and central adjudication of MACE in at-risk patients with stable coronary disease (24).

Only women with postmenopausal osteoporosis were evaluated in our study. Differences exist in cardiovascular disease biology between women and men. For example, women experience HF with preserved ejection fraction more frequently as a result of hypertension and/or diabetes, while HF with reduced ejection fraction from prior ischemic events is more frequent in men (25-29). Thus, sex-specific consideration of cardiovascular responses to abaloparatide were not encompassed by this study.

## Conclusions

Treatment with abaloparatide and teriparatide resulted in a transient increase in HR and a small decrease in 1-hour postdose BP. These changes were not associated with an increase in serious cardiac AEs evaluated in ACTIVE, MACE, or HF in postmenopausal women with osteoporosis.

## Data Availability

Restrictions apply to the availability of data generated or analyzed during this study to preserve patient confidentiality or because they were used under license. The corresponding author will, on request, detail the restrictions and any conditions under which access to some data may be provided.

## Abbreviations

ACTIVE: Abaloparatide Comparator Trial in Vertebral Endpoints
AE: adverse event
BP: blood pressure
bpm: beats per minute
CI: confidence interval
ECG: electrocardiogram
FDA: US Food and Drug Administration
HF: heart failure
HR: heart rate
MACE: major adverse cardiovascular event
MedDRA: medical dictionary for regulatory activities
PTH: parathyroid hormone
PTHR1: PTH type 1 receptor
PTHrP: PTH-related peptide
SMQs: standardized MedDRA queries

## Additional Information

***Disclosure Summary:*** F.C. has received institutional grants and research support from Amgen and Eli Lilly; has served as a consultant for Amgen, Eli Lilly, Merck, Radius Health, Inc. (Radius), and Tarsa; has served on the speakers’ bureaus for Amgen, Eli Lilly, and Radius; and has served on advisory boards for Amgen, Eli Lilly, Merck, and Radius. L.R.P. has received research funding from the National Institutes of Health (NIH) and the Barnes-Jewish Hospital Foundation; has consulted for Radius Health, Inc; and has stock in Exact Sciences, Medtronic, Johnson and Johnson, and Seattle Genetics. D.A.T. has received research funding from the NIH and from the American Diabetes Association. Y.W. and B.M. are employees of and hold equity in Radius Health, Inc. S.R.C. is a consultant to and has received grant support from Amgen and is a consultant to Radius Health, Inc.
